# Impact of Virtual Reality Headset on Pain and Anxiety for Bedside Abdominal VAC Dressing Change (VIRPA): A Randomized Controlled Clinical Trial

**DOI:** 10.1002/hsr2.71877

**Published:** 2026-02-22

**Authors:** Beatriz Barberá‐Carbonell, Amaniel Kefleyesus, Reza Djafarrian, Sandrine Geinoz, Dieter Hahnloser, Fabian Grass, Martin Hübner

**Affiliations:** ^1^ Department of Visceral Surgery, University Hospital Lausanne (CHUV) University of Lausanne (UNIL) Lausanne Switzerland; ^2^ Department of General Surgery GHOL Nyon Hospital Nyon Switzerland

**Keywords:** anxiety, pain, protocol, satisfaction, virtual reality headset

## Abstract

**Background and Aims:**

Virtual reality (VR) distraction techniques are promising adjuncts to reduce pain and anxiety. This study assessed the impact of VR distraction during bedside change of vacuum assisted closure (VAC) dressings.

**Methods:**

In this non‐blinded randomized superiority trial, patients scheduled for bedside change of a subcutaneous VAC dressing were allocated to receive distraction through VR masks in addition to a standardized pain protocol (intervention) or pain protocol alone (control). Primary endpoints were pain scores assessed by a visual analogue scale (VAS: 0–10), secondary outcomes were anxiety (State Trait Anxiety Inventory (STAI), VAS: 0–10), hemodynamic parameters, and satisfaction (VAS: 0–10).

**Results:**

Pre‐ and postoperative pain levels were 2.2 ± 2.2 versus 2.0 ± 2.1 (*p* = 0.38) for the intervention group (21 patients) compared with 2.6 ± 2.1 versus 2.2 ± 1.6 (*p* = 0.26) for the control group (19 patients), with no significant difference between the groups (*p* = 0.38). No differences between the two groups were observed for blood pressure and heart rate (HR), besides lower post‐procedural HR in the intervention group. Anxiety was reduced in both groups post VAC change in the intervention and control group: STAI 40 ± 12 pre‐VAC versus 30 ± 8 post VAC and 45 ± 14 pre‐VAC versus 32 ± 9 post VAC (both *p* < 0.01), ∆VAS −2 (interquartile range IQR 0, −3) versus −2 (IQR 0, −5), both *p* < 0.01. Postinterventional satisfaction was 8.3 ± 1.9 (intervention) versus 7.5 ± 2.4 (*p* = 0.11).

**Conclusion:**

Pain and anxiety were well managed within a standardized pain protocol, with or without adjunct VR distraction. While this may be due to limited statistical power in this exploratory pilot study, further studies should focus on patients with insufficient control of pain and anxiety with a standard protocol to assess the additional value of VR distraction.

**Trial Registration:** study registered in the FOPH portal SNCTP (Swiss National Clinical Trial): NCT04472416.

**Abbreviations**
ERP: enhanced recovery pathway
VAC: vacuum assisted
VAS: visual analogue scale
VR: virtual reality

## 1 Introduction

Enhanced recovery pathways (ERP) have improved postsurgical recovery and outcomes through a standardized array of perioperative measures [1, 2]. Opioid‐sparing analgesia as an ERP cornerstone represents an ongoing challenge, with inconsistent results of the suggested multimodal approach [3].

Adjunct analgesics and alternative methods have recently gained interest [4]. Virtual reality (VR) headsets may help to reduce anxiety and pain and to increase patient comfort. Both active and passive (immersive) VR modalities have been described [5]. A beneficial impact of VR distraction has particularly been shown in specific populations such as burn victims [6] or patients undergoing dental procedures [7], children undergoing venipuncture [8], or patients undergoing endoscopic procedures [9]. However, evidence remains scarce for general surgery.

Vacuum Assisted Closure (VAC) dressings are used as a routine practice for abdominal wounds to accelerate the healing process [10] and to prevent wound infections [11]. Depending on the extent of the wound, dressing changes can be performed under general anesthesia or in a bedside setting, which, however, may cause anxiety and stress for the patient. Hence, there may be a particular benefit of VR distraction in the setting of VAC dressing changes, potentially limiting the need for repeated general anesthesia and simplifying logistic processes.

To date, no studies have assessed the added value of VR distraction in this setting. To address this shortcoming, the aim of the present study was to measure the impact of VR distraction in addition to a standardized pain protocol during bedside VAC dressing changes, with the hypothesis to decrease pain and anxiety and to increase patient comfort and satisfaction.

## 2 Methods Trial Design

This trial was designed as a prospective, non‐blinded, randomized controlled trial (1:1) of VR in adjunction to a standardized analgesic protocol versus standardized analgesic protocol alone.

Patients were enrolled at one study site in Switzerland (Visceral Surgery department at Lausanne University Hospital, CHUV) from April 1, 2021, to September 30, 2023.

The report from this trial follows the CONSORT statement [12].

### 2.1 Participants

Inclusion criteria were: patients ≥ 18 years old, requiring a bedside abdominal VAC dressing change, able to give informed consent.

Exclusion criteria were patients with impaired cognitive status and unable to follow the study procedures due to language. Patients with a prior inclusion in the same trial (only the first VAC procedure) and pregnant patients were excluded.

### 2.2 Intervention

Each patient received standardized local and systemic analgesia 30 min before starting the procedure (Supporting Information S1: Appendix 1). Benzodiazepines were administered upon request.

The virtual reality device (VRD, Healthy Mind company) was activated 15 min before the beginning of the procedure and used until 15 min after finishing the wound dressing. This VRD is a medical solution that is meant to transport the user/patient into purposely designed 3D experiences to journey through 9 different natural and therapeutic environments of his/her choice (e.g., Japanese gardens, mountains, beaches, etc.). The solution intends to use the brain's cognitive abilities to modulate pain pathways through visual and auditory stimulation, attention diversion, and various advanced psychological principles to reduce pain and anxiety. These immersions are amplified by the realism of 3D environments projected on the headset; a soothing sound atmosphere broadcasted via headphones along with specific medical hypnosis scripts for each theme. More specifically, music composed specifically for each environment intends to offer a multi‐sensory experience. Targeted breathing exercises are also integrated for better anxiety management in cardiac coherence or deep breathing for post‐operative rehabilitation.

In case of pain or discomfort, patients could communicate at any time directly with the caregiver. Further metrics, including patient immersion, engagement, or preference, were not assessed in the setting of this study.

The sequence of the intervention is illustrated in the Supporting Information S2: Appendix 2.

### 2.3 Outcomes

The primary outcome was pain assessed by a visual analogue scale (VAS) before and after the VAC change.

Secondary outcomes were anxiety assessed using the validated questionnaire State Trait Anxiety Inventory (STAI‐Y) [13] and a VAS for anxiety (0–10) before and after the procedure. Overall satisfaction was assessed through VAS (0–10) after the procedure. Hemodynamic parameters were measured before and after the procedure to assess clinical response correlated to pain and anxiety.

### 2.4 Statistical Analysis

Sample size estimation was based on the effect estimated from previous VR studies [14, 15] using pain as the primary endpoint. A sample size of 16 participants per group would enable a two‐arm RCT to detect a between‐group VAS difference of three with a pooled standard deviation of ±3 with 80% power at 5% level of significance. To account for potential dropouts, 40 participants were recruited overall.

Eligible participants were randomly assigned in a 1:1 ratio to the intervention or control group by computer generation using Redcap (Research Electronic Data Capture database). Data were prospectively gathered and computed by two authors (B.B.C. and R.D.).

Outcomes were analyzed on an intention‐to‐treat basis. Univariate analysis was performed with Mann–Whitney U‐test or Student *t*‐test for continuous variables and Fischer's exact test for categorical variables. A *p* value of < 0.05 was considered statistically significant, and all tests were 2‐sided. Between‐group differences, 95% confidence intervals, and effect sizes (Cohen's *d*) were calculated to quantify the magnitude and precision of observed effects. A per‐protocol sensitivity analysis, restricted to VR participants with ≥ 60 min of exposure, was performed to assess robustness. Given the modest sample size, a post‐hoc power calculation was conducted for the primary endpoint. Detailed effect estimates and sensitivity analyses are provided in the Supporting Information.

Data analysis was performed with the Statistical Software for the Social Sciences SPSS Advanced Statistics 29 (IBM Software Group, 200W. Madison St., Chicago, IL; 60606 USA) and GraphPad Prism 10 (GraphPad Software Inc., La Jolla, CA, USA).

### 2.5 Ethical Aspects

This trial was approved by the local ethics committee (ID 2020‐00091) and was registered in the FOPH (Federal Office of Public Health) portal SNCTP (Swiss National Clinical Trial) with the ID SNCTP000004166 and in clinicaltrials.gov (ID NCT04472416). All participants provided written informed consent for study participation.

## 3 Results

Ninety‐six patients were screened for eligibility, forty‐two were randomized, and 19 (intervention) and 21 (control) received the allocated intervention (Supporting Information S3: Appendix 3). Indications for VAC dressings were surgical site infection (SSI, 17 patients), while the remaining patients had preventive VACs after contaminated surgery according to institutional protocol (*n* = 23, no differences between groups). Both groups were comparable regarding age (61 ± 11 vs. 62 ± 17), sex (female: 9 vs. 13), American Society of Anesthesiologists (ASA) scores (≥ 3: 18 vs. 18, all non‐significant), and wound size (median 10 cm in both groups). Three patients (2 in the control group and 1 in the intervention one) needed an adaptation of the systemic analgesic protocol considering the use of other pain strategies during the procedure. Six patients in the intervention group had a duration of VR of less than 60 min.

**Figure 1:**
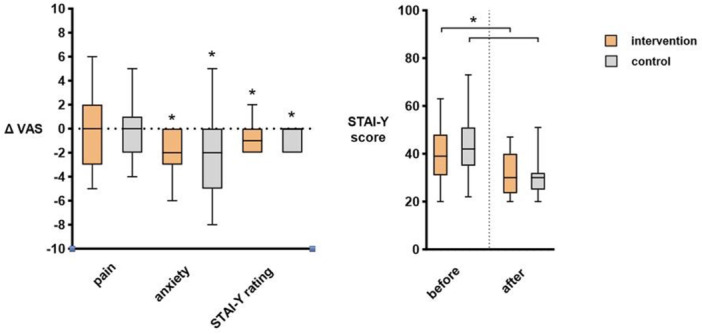
Pain and anxiety scores. Vertical box plots illustrating delta values (∆) for intervention and control groups. The left graph illustrates delta values, whereas the right graph represents STAI‐Y scores. STAI‐Y, state‐trait anxiety inventory; VAS, visual analogue scale. * indicate statistical significance (*p* < 0.05).

**Figure 2:**
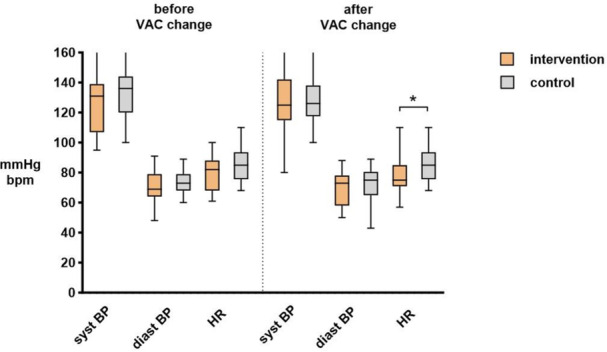
Hemodynamics. Vertical box plots illustrating pre‐ and post‐intervention values for both groups (intervention vs. control). bpm, beats per minute; BP, blood pressure; diast, diastolic; HR, heart rate; syst, systolic; VAC, vacuum‐assisted closure. * indicates statistical significance (*p* < 0.05).

Pre‐ and postoperative pain levels were 2.2 ± 2.2 versus 2.0 ± 2.1 (*p *= 0.38) for the intervention group compared with 2.6 ± 2.1 versus 2.1 ± 1.6 (*p *= 0.26), with no significant difference between the groups (*p *= 0.38) as displayed in Figure 1. Similarly, no statistically significant differences were noted for pre‐ and postinterventional blood pressure and heart rate (Figure 2). Anxiety was reduced in both groups post VAC change in the intervention and control group: STAI 40 ± 12 pre‐VAC versus 30 ± 8 post VAC and 45 ± 14 pre‐VAC versus 32 ± 9 post VAC (both *p* < 0.01), ∆VAS −2 (interquartile range IQR 0, −3) versus −2 (IQR 0, −5), both *p* < 0.01. Postinterventional satisfaction was 8.3 ± 1.9 (intervention) versus 7.5 ± 2.4 (control, *p* = 0.11).

Effect‐size estimates, confidence intervals, and sensitivity analyses restricted to participants who received ≥ 60 min of VR exposure yielded results consistent with the primary analysis, indicating no clinically meaningful impact of the intervention (Tables S1 and S2). A post‐hoc power assessment showed that the detectable effect size for 80% power exceeded the effect actually observed, suggesting that the study was underpowered to identify small differences (Table S3). Taken together, these results demonstrate that the VR intervention did not meaningfully alter pain, anxiety, or satisfaction within the available sample size.

## 4 Discussion

Pain and anxiety during bedside VAC dressing changes were equally well controlled within a standardized pain protocol with or without a VR headset. Satisfaction was high in both comparative groups.

Some studies had shown a significant reduction of pain levels with the adjunction of VR [6, 8, 14, 15, 16]. However, settings were very specific, including range‐of‐motion physical therapy in burn patients [6], children aged 4–12 years undergoing venipuncture [8], young adults experiencing ischemic pain created with a blood pressure instrument [14], or patients undergoing wound care and dressing changes after hemorrhoidectomy [15]. In all these settings, pain scores were significantly lower in the VR groups, while no impact on HR was revealed in the latter study [15]. Especially burn victims may benefit from passive VR distraction as an adjunct analgesia technique in the setting of repeated and painful dressing changes, potentially revealing a more significant impact of VR techniques compared to bedside VAC changes in this study [6, 17]. Since in the present study baseline pain scores were already low, the projected 3‐point difference in VAS scores used for the power calculation may have been overly optimistic, as suggested by the post‐hoc power calculation (Table S3). Interestingly, the present study observed a decreased post‐procedural HR in the intervention group, which may represent a surrogate finding for lower pain or anxiety related to VR distraction. A further study focusing on patients undergoing burn dressing changes revealed another interesting aspect [18]. In their study, the benefit of VR distraction appeared to be related to additional interaction through active participation in specifically designed VR games. This contrasts with the passive distraction strategy used in this present study.

Other studies observed no significant additional benefit of VR distraction. However, self‐evaluation questionnaires revealed reduced discomfort during dental procedures [7]. Interestingly, VR distraction devices improved satisfaction and decreased anxiety and pain only among patients with a high pre‐procedural anxiety score in patients undergoing colonoscopy [9]. VR distraction may hence be particularly beneficial in this group of patients with high pre‐procedural anxiety or pain, a finding which may have contributed to the negative results of the present study given pain scores were very low throughout. Future studies may have to focus on procedures with higher baseline pain scores to detect a more significant impact. However, the small sample size of our study unfortunately impeded further subgroup analysis.

Based on our clinical experience, a systemic and local pain protocol was established in collaboration with anesthetists, including the use of benzodiazepines. While indications and procedures were different in former studies, a specific analgesic protocol was not always administered [8, 9, 14], reported [7, 15], or individualized [6], impeding uncritical comparison of findings. However, the use of benzodiazepines may blunt to some extent both pain and anxiety perception, thereby potentially reducing the detectable effect of the intervention.

Anxiety was significantly reduced pre‐ and post‐intervention in both comparative groups, however without difference between the groups. Anxiety was reduced through VR in several former studies [7, 8, 9]. Again, the small sample size in the present study impeded subgroup analysis to confirm these findings. Given VR represents a low‐cost and low‐risk adjunct treatment, which can be easily implemented in routine clinical practice, it may be particularly useful in patients susceptive for alternate therapies.

The present study revealed no hemodynamic differences between the two groups, which coincides with the results of other studies [7, 8, 15, 16]. Again, the very specific settings of these former studies impede more detailed comparisons. Satisfaction was higher in the intervention group, albeit not significantly. Larger studies are needed to confirm these preliminary findings. Further studies should probably focus on specific target populations, including patients with a specific interest in alternative therapies, patients with higher baseline pain or anxiety scores, and more complex clinical settings and procedures, to potentially reveal a more significant impact of VR distraction.

Several limitations need to be addressed. The estimated effect may have been overly optimistic due to low baseline pain scores in the setting of the chosen procedure, and the study sample in consequence too small to detect small effects (type II error). The small sample size also impeded further subgroup (i.e., higher baseline anxiety), per‐protocol or sensitivity analyses to further help interpreting treatment effects. Further studies should therefore focus on larger cohorts of patients who have insufficient control of symptoms (pain and/or anxiety) with standard means and who show a high interest in alternative therapies. Long recruitment time was related to the pandemic situation and the impossibility of using the device for a long period to avoid virus transmission. Few protocol deviations regarding additional use of systemic pain medication (two in the control group and one in the intervention group) and VR use below the anticipated 60 min in six patients must be emphasized. In the VR distraction group, patient immersion, engagement, or preference were not assessed in the setting of this study. However, they may have contributed to the null findings. The study design was non‐blinded, which may introduce performance and detection bias.

The strength of the present study is related to its multi‐component assessment (subjective and objective parameters).

## 5 Conclusions

A VR headset had no measurable benefit in patients undergoing bedside VAC dressing change with a standardized pain protocol in the present study. A potential explanation may be related to the efficient institutional pain management protocol with very low pain scores throughout. Further studies should focus on patients with insufficient control of pain and anxiety with a standard protocol to assess the additional value of VR distraction. However, the low‐cost and low‐risk adjunct treatment was nevertheless very well received by patients and staff and deserves future evaluation with a focus on more complex clinical settings and patients with a specific interest for alternative treatments.

## Author Contributions

**Beatriz Barberá‐Carbonell:** conceptualization, formal analysis, investigation, methodology, writing – original draft. **Amaniel Kefleyesus:** formal analysis, investigation, methodolgy, writing – review and editing. **Reza Djafarrian:** conceptualization, investigation, software, writing – review and editing. **Sandrine Geinoz:** formal analysis, investigation, methodology, resources, writing – review and editing. **Dieter Hahnloser:** conceptualization, supervision, writing – review and editing. **Fabian Grass:** formal analysis, investigation, methodology, writing – original draft. **Martin Hübner:** conceptualization, data curation, formal analysis, investigation, methodology, software, supervision, writing – review and editing.

## Funding

The authors received no specific funding for this work.

## Disclosure

This study was presented at the Annual Swiss Surgical Meeting 2024 on May 26–29, 2024, Davos, Switzerland.

## Conflicts of Interest

The authors declare no conflicts of interest.

## Transparency Statement

The corresponding author, Martin Hübner, affirms that this manuscript is an honest, accurate, and transparent account of the study being reported; that no important aspects of the study have been omitted; and that any discrepancies from the study as planned (and, if relevant, registered) have been explained.

## Supporting information


**Online appendix 1:** Standardized local and systemic analgesia protocol.

**Online appendix 2:** Virtual reality distraction. Abbreviations: STAI, state‐trait anxiety inventory; VAS, visual analogue scale; VAC, vacuum‐assisted closure; VR, virtual reality.

**Online appendix 3:** Flow chart. Abbreviations: GA, general anesthesia; SAP, standard analgesic protocol; VR, virtual reality; VRD, virtual reality device.

**Supplementary table S1:** Between group differences (ITT population).

**Supplementary table S2:** Between group differences (PP population).

**Supplementary table S3:** Post‐hoc power analysis for the primary outcome.

## Data Availability

Data is available upon request.
