# Supplementary figures and images for: Impact of Virtual Reality Headset on Pain and Anxiety for Bedside Abdominal VAC Dressing Change (VIRPA): A Randomized Controlled Clinical Trial
Source: Health Sci Rep. 2026 Feb 22;9(2):e71877. doi: 10.1002/hsr2.71877 (PMC12927986; doi:10.1002/hsr2.71877)

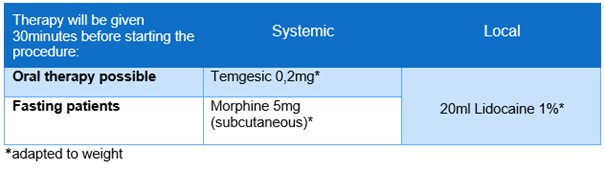

Supplement: Supplementary file 1 — Online appendix 1: Standardized local and systemic analgesia protocol. [file HSR2-9-e71877-s006.jpg]

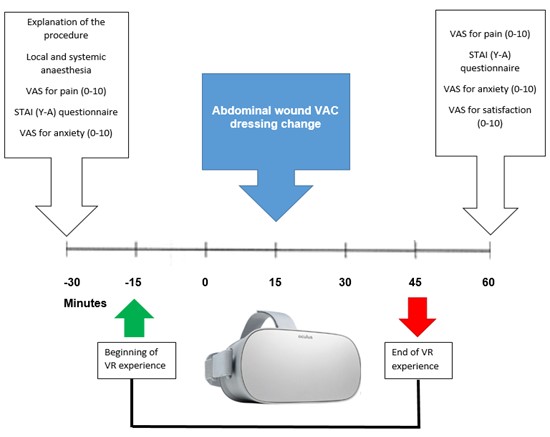

Supplement: Supplementary file 2 — Online appendix 2: Virtual reality distraction. Abbreviations: STAI, state‐trait anxiety inventory; VAS, visual analogue scale; VAC, vacuum‐assisted closure; VR, virtual reality. [file HSR2-9-e71877-s005.jpg]

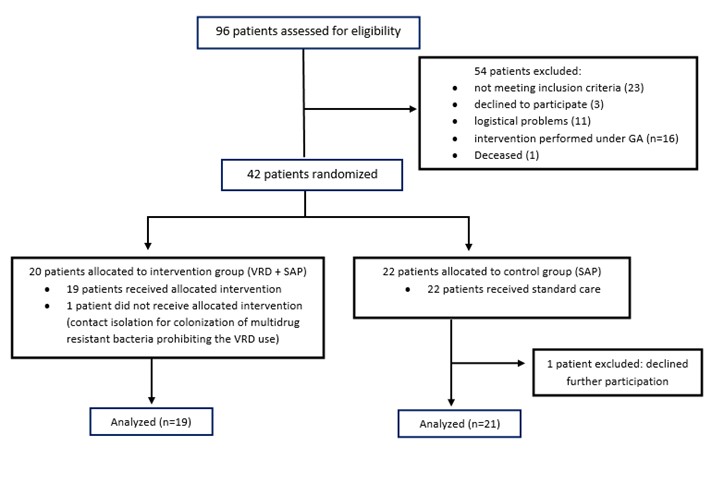

Supplement: Supplementary file 3 — Online appendix 3: Flow chart. Abbreviations: GA, general anesthesia; SAP, standard analgesic protocol; VR, virtual reality; VRD, virtual reality device. [file HSR2-9-e71877-s004.jpg]
