# Supplementary material for: Impact of Virtual Reality Headset on Pain and Anxiety for Bedside Abdominal VAC Dressing Change (VIRPA): A Randomized Controlled Clinical Trial
Source: Health Sci Rep. 2026 Feb 22;9(2):e71877. doi: 10.1002/hsr2.71877 (PMC12927986; doi:10.1002/hsr2.71877)
Supplement: Supplementary file 4 — Supplementary table S1: Between group differences (ITT population). [file HSR2-9-e71877-s002.docx]

# Table S1. Between-group differences (ITT population)

| Outcome | VR (mean ± SD) | Control (mean ± SD) | Mean diff VR–Control (95% CI) | p-value | Cohen's d |
| --- | --- | --- | --- | --- | --- |
| Pre-procedural pain (VAS) | 2.2 ± 2.2 | 2.6 ± 2.1 | -0.4 (-1.7; 1.0) | 0.605 | -0.17 |
| Post-procedural pain (VAS) | 2.0 ± 2.1 | 2.2 ± 1.6 | -0.2 (-1.4; 1.0) | 0.751 | -0.10 |
| Pre-procedural anxiety (STAI-Y) | 39.7 ± 12.1 | 44.7 ± 14.1 | -5.0 (-13.1; 3.1) | 0.236 | -0.38 |
| Post-procedural anxiety (STAI-Y) | 29.6 ± 7.9 | 31.6 ± 8.7 | -2.0 (-7.1; 3.1) | 0.451 | -0.24 |
| Post-procedural satisfaction (0–10) | 8.3 ± 1.9 | 7.5 ± 2.4 | 0.8 (-0.5; 2.2) | 0.111 | 0.39 |
