# Supplementary material for: Impact of Virtual Reality Headset on Pain and Anxiety for Bedside Abdominal VAC Dressing Change (VIRPA): A Randomized Controlled Clinical Trial
Source: Health Sci Rep. 2026 Feb 22;9(2):e71877. doi: 10.1002/hsr2.71877 (PMC12927986; doi:10.1002/hsr2.71877)
Supplement: Supplementary file 5 — Supplementary table S2: Between group differences (PP population). [file HSR2-9-e71877-s001.docx]

# Table S2. Between-group differences (per-protocol population)

| Outcome | VR (mean ± SD) | Control (mean ± SD) | Mean diff VR–Control (95% CI) | p-value | Cohen's d |
| --- | --- | --- | --- | --- | --- |
| Pre-procedural pain (VAS) | 2.5 ± 2.5 | 2.6 ± 2.1 | -0.1 (-2.0; 1.9) | 0.944 | -0.03 |
| Post-procedural pain (VAS) | 3.0 ± 2.5 | 2.2 ± 1.6 | 0.8 (-1.1; 2.7) | 0.418 | 0.43 |
| Pre-procedural anxiety (STAI-Y) | 41.6 ± 9.3 | 44.7 ± 14.1 | -3.0 (-11.9; 5.8) | 0.507 | -0.23 |
| Post-procedural anxiety (STAI-Y) | 31.8 ± 9.3 | 31.6 ± 8.7 | 0.2 (-7.3; 7.6) | 0.963 | 0.02 |
| Post-procedural satisfaction (0–10) | 7.9 ± 2.0 | 7.5 ± 2.4 | 0.4 (-1.3; 2.1) | 0.651 | 0.18 |
