# Supplementary material for: Impact of Virtual Reality Headset on Pain and Anxiety for Bedside Abdominal VAC Dressing Change (VIRPA): A Randomized Controlled Clinical Trial
Source: Health Sci Rep. 2026 Feb 22;9(2):e71877. doi: 10.1002/hsr2.71877 (PMC12927986; doi:10.1002/hsr2.71877)
Supplement: Supplementary file 6 — Supplementary table S3: Post‐hoc power analysis for the primary outcome. [file HSR2-9-e71877-s003.docx]

# Table S3. Post-hoc power analysis for the primary outcome

| Outcome | n_VR | n_Control | Cohen's d (observed) | Observed power (α = 0.05) | d for 80% power | Min. detectable diff (VAS) |
| --- | --- | --- | --- | --- | --- | --- |
| Post-procedural pain (VAS) | 19 | 21 | -0.10 | 0.06 | 0.91 | 1.7 |
